# A general approach to compensation for losses incurred due to public health interventions in the infectious disease context

**DOI:** 10.1007/s40592-020-00104-2

**Published:** 2020-03-04

**Authors:** Søren Holm

**Affiliations:** 1 Centre for Social Ethics and Policy, School of Law, University of Manchester, Manchester, M13 9PL UK; 2 Center for Medical Ethics, Faculty of Medicine, HELSAM, University of Oslo, Oslo, Norway

**Keywords:** Compensation, Quarantine, Isolation, Vaccination, Public health ethics, Infectious disease

## Abstract

This paper develops a general approach to how society should compensate for losses that individuals incur due to public health interventions aimed at controlling the spread of infectious diseases. The paper falls in three parts. The first part provides an initial introduction to the issues and briefly outlines five different kinds of public health interventions that will be used as test cases. They are all directed at individuals and aimed at controlling the spread of infectious diseases (1) isolation, (2) quarantine, (3) recommended voluntary social distancing, (4) changes in health care provision for asymptomatic carriers of multi-resistant microorganisms, and (5) vaccination. The interventions will be briefly described including the various risks, burdens and harms individuals who are subject to these interventions may incur. The second part briefly surveys current compensation mechanisms as far as any exist and argue that even where they exist they are clearly insufficient and do not provide adequate compensation. The third part will then develop a general framework for compensation for losses incurred due to public health interventions in the infectious disease context. This is the major analytical and constructive part of the paper. It first analyses pragmatic and ethical arguments supporting the existence of an obligation on the part of the state to compensate for such losses, and then considers whether this obligation can be defeated by (1) resource considerations, or (2) issues relating to personal responsibility.

## Introduction

The control of the spread of infectious diseases is an important aim of public health activities. At the global level it is important to control epidemics of new and emerging diseases e.g. Ebola, SARS, MERS-CoV, Covid-19,^1^ as well as the spread of new variants of well-known diseases e.g. influenza, or the spread of old diseases in new geographic locations e.g. Zika virus and Dengue fever. These diseases lead to mortality, morbidity, potential loss of social cohesion and significant economic losses to individuals and society. At a more local level it can be equally important to prevent the spread of antibiotic resistant bacteria or their entry into specific hospital wards, or to quell localised outbreaks of particular diseases, e.g. meningitis among university students at the start of a new academic year.

However, many of the tried and tested public health interventions that are used to control the spread of infectious diseases creates financial and non-financial costs and burdens for the individuals that are subject to these interventions. Some interventions impose such costs and burdens on anyone who is subject to them, while others creates a risk of rare, but very significant harm. The purpose of this paper is to develop a general ethical framework for compensation for these costs and burdens. In order to develop such a framework we have to answer two questions:

1. Under what circumstances does society^2^ have an obligation to provide compensation?
2. What can defeat an obligation identified in 1?

Throughout the analysis it will be assumed (a) that the public health intervention is legitimate, i.e. that it is justified both ethically and legally in the specific context, (b) that it is well designed and well delivered, and (c) that there are separate, effective mechanisms for compensation for costs and burdens that arise as a result of error, fault or negligence.^3^ Or to put it differently, the analysis is aimed at answering under what conditions there ought to be no-fault compensation for costs and burdens in the context of legitimate and competently delivered public health interventions. In relation to interventions that include an element of a *prima facie* rights violation, the assumption of legitimacy entails that any rights violation is justified. The analysis will bracket the question of whether there ought to be specific redress for such justified rights violations in themselves, irrespective of whether they cause harms, burdens or costs. There is an extant literature on compensation for costs created by public health interventions but it is limited and not generally well referenced or integrated in the broader literature on the ethics of public health interventions (Markovits 2005; Rothstein and Talbott 2007a; b; Holm 2009; Selgelid 2009; Ly et al 2009; Silva et al. 2016).

The analysis will use five different kinds of public health interventions as test cases to cover a wide spectrum of possible losses. These interventions are used because each raise slightly different issues in relation to compensation. Each could be analysed in much greater detail than is possible in this paper that aims at developing and justifying a general framework for compensation:

1. Isolation of infected individuals,
2. quarantine of potentially infected individuals,
3. recommended voluntary social distancing/self-isolation,
4. changes in health care provision for asymptomatic carriers of multi-resistant microorganisms, and
5. vaccination.

Isolation, quarantine and voluntary social distancing may all cause a loss of earnings for individuals if employers are not willing to continue paying wages for the period when the person is not able to work because of the public health intervention.^4^ They may also cause more long-term losses if the employment status of those who are subject to these interventions is not protected and employers dismiss them due to their absence from work. Those who are self-employed will also in many cases experience a direct loss of income. Costs may also accrue to the family of a person who is a subject of isolation etc. if the family has to reorganise caring or family work commitments due to the absence of the person. It is well documented that these financial costs are real, that they are of concern to people, and that they may lead to non-compliance with the intervention as a whole or elements of it (Blendon et al 2006; Baum et al 2009; Blake et al 2010; Kavanagh et al 2012). Quarantine, and social distancing measures involving households with both infected and uninfected members also potentially creates an increased risk of infection for people who are not infected when put in quarantine or complying with social distancing advice, but who are forced by the intervention to be in close contact with potential or known infected and infectious persons. This has been dramatically illustrated by the quarantine of the Diamond Princess Cruise liner in the Japanese port of Yokohama during the Covid-19 outbreak. At the time of writing more than 600 cases of Covid-19 infection has been identified on the ship at the end of 14 days of quarantine, and it is very likely that most of the transmission has happened during the quarantine (BBC 2020). The countries that are re-patriating ostensibly unaffected citizens from the ship are putting them into 14 days of further quarantine on arrival (BBC 2020). The Diamond Princess quarantine event also illustrates the social justice issues that may occur during quarantine. The passengers were quarantined in their own rooms with en-suite bathrooms, whereas the ship’s crew continued to live two to a room with shared bathrooms and toilets (McCurry 2020).

Patients who are asymptomatic carriers of multi-resistant microorganisms will often experience significant changes in their health care provision.^5^ The changes will depend on the specific microorganism in question and the scope of this paper does not allow us to survey the whole variety of possible implications of carrier status. But we will look at one example, i.e. the treatment of patients known to be carriers of Methicillin Resistant Staphylococcus Aureus (MRSA) since this is a fairly common condition. Patients diagnosed as long term MRSA carriers after unsuccessful attempts of de-colonising will have an ‘MRSA flag’ added to their notes. This may affect the scheduling of elective procedures, and they will at every subsequent admission be isolated in a single room or in a ward with other MRSA carriers with an isolation notice on the door. They can receive visitors, but not leave the room. The number of health care professionals attending to them will be minimised, and special precautions will be taken if they have to go for X-rays or other diagnostic procedures outside of the ward. Any physiotherapy, blood sampling etc. will take place at the end of the therapists’ or phlebotomists’ rounds to minimise the risk of spreading MRSA (King’s College Hospital 2013). When visiting out-patient clinics known MRSA carriers will be booked in the last slots of the day, or in some cases after the normal clinics have ended to prevent transmission in the waiting room. MRSA carrier status thus does not impose any direct costs, but it does create significant burdens when carriers need health care, starting with the simple act of having to declare carrier status when getting into contact with a new health care setting. The publicly observable effects of carrier status may also lead to stigmatisation and loss of self-worth, and these social and psychological effects may even ‘infect’ non-carriers in the same household (Ploug et al 2015; Fynbo and Jensen 2018).

Vaccination is one of the safest public health interventions, with extremely low rates of serious side-effects. But even though the risk is extremely low some people will still end up suffering very significant harm which is directly caused by the vaccination. All vaccines containing live attenuated microorganisms can in rare cases cause vaccine induced disease, and other vaccines can cause serious side-effects due to immune cross-reactions (for a comprehensive listing of known adverse vaccine reactions see Appendix 1 in WHO 2014). These harms are randomly distributed in the sense that there is no link between the likely individual protection achieved by vaccination and the harm eventuating. We will never know whether the person who is harmed would at some point in the future have needed the protection provided by the vaccination.

## Current compensation mechanisms

The fact that some public health interventions may cause harm has long been recognised, especially in relation to vaccination and a number of countries have compensation schemes for vaccine injuries (Looker and Kelly 2011). These schemes exist both in countries with mandatory vaccination and in countries with purely voluntary vaccination. But, they are much more common in high-income countries then in low-income countries. A recent paper found that of the 62 countries that have mandatory vaccination programs only seven have no-fault compensation schemes, and all of those seven countries were high-income countries (Attwell et al. 2019).

The schemes typically cover well known severe side-effects such as vaccine induced polio or other vaccine related infections and also any new severe side-effects. Those affected do not have to show error or negligence on the part of the health care professionals delivering the vaccination or the manufacturers of the vaccine, but they have to show that their injury was caused by the vaccination with the precise standard for causation differing in different legal systems and often mirroring the local standard for causation in negligence claims. Some compensation schemes for vaccine injury have an upper limit of the amount of compensation it is possible to receive. In the UK the limit is for instance currently GBP 120,000 or approximately 155,000$ US dollars. This is far less than the amount of compensation awarded by the courts if the same level of harm had been caused by negligence. Some schemes are pre-emptive in the sense that if you apply to the scheme you cannot at the same time or later sue the manufacturers or health care professionals, whereas others allow a claimant to pursue both routes.

Even though the schemes are supposed to provide easier and faster access to compensation for people suffering vaccine injuries than normal legal processes in the courts they can never the less be slow and difficult to navigate (Henson 2007), and there are also documented instances where governments try to avoid or minimise pay-outs by exploiting legal technicalities. In an English case involving a boy who had developed severe narcolepsy with cataplexy after receiving a pandemic flu vaccine the government for instance argued that his disability did not reach the required level of severity for compensation at the time of assessment when he was in school and living at home with his parents. Compensation was therefore withheld, even though it was agreed that his disability would cross the threshold when he grew up and would have to live independently. Fortunately for the family the Court of Appeal rejected this bit of legal sophistry (Secretary of State for Work and Pensions V FG On Behalf of John (A Minor) [2017] EWCA Civ 61).

Compensation schemes for losses caused by isolation, quarantine and social distancing are less common and can mainly be found in countries that were significantly affected by the SARS outbreak in 2003 (e.g. Canada, China, Hong Kong, Singapore, Taiwan) (Rothstein and Talbott 2007b) and the MERS-CoV outbreak in 2015–2016, although they were also in place in most Australian states prior to SARS (Ly et al. 2009). In relation to the SARS outbreak the most discussed piece of legislation is the Ontario ‘SARS Assistance and Recovery Act 2003′ which gave employees who were quarantined a legally protected right to “leave of absence without pay” and to reinstatement in their previously held position. As a matter of policy Ontario also instituted an economic assistance program which was later followed by a Federal Canadian program offering flat rate compensation of CAD 400 per week of quarantine to full time workers and CAD 200 to part time workers, with an overall cap of CAD 6,000 (SARS Commission 2005). A small number of US states also introduced laws following the SARS outbreak that in various ways protect the employment status of persons who are isolated or quarantined, and this was also proposed at the Federal level, but the Federal law was never passed (Rothstein and Talbott 2007a; b).

The most recent round of legislation occurred during the MERS-CoV outbreak in 2015–2016 where South Korea introduced a comprehensive compensation scheme which covered a variety of losses (Infectious Disease Control and Prevention Act, Act No. 14286, Dec 2, 2016). Kim describes it in the following way (Kim 2016):

A salient example is the government compensation policy for those who were ordered to stay home to prevent transmission of the disease, and compensation for the funeral costs to the surviving families of the deceased. Although the government was not able to have a public discussion of the appropriate levels of compensation in advance, the decisions about compensation and the procurement of financial resources for compensation were made in the middle of the outbreak, and were motivated by the discovery that those who were quarantined were faced with loss of income and employment, putting their livelihood at stake. Food and basic necessities were provided during the period of quarantine, and financial compensation for the loss of income was subsequently made according to the size of the family. In July 2015, the Infectious Diseases Prevention Act in Korea was revised to include clauses for the compensation of financial losses to those who were placed under quarantine, the hospitals that provided medical care for MERS-CoV patients, and the surviving family members of those who died from MERS-CoV.

This brief survey of existing compensation schemes show that such schemes are not widespread and that even where they exist they are often of limited scope and often difficult to access. In most cases they do not compensate everyone with a legitimate claim, and do not compensate those who get compensation adequately.

## The basis for an obligation to compensate

There are two main types of argument supporting an obligation for the state to compensate for losses created by public health interventions. The first type are pragmatic arguments and the second ethical arguments, but as we shall see this is one of the cases where ‘all roads lead to Rome’ in the sense that we get strong support for an obligation to compensate from both pragmatic and ethical arguments, and from ethical arguments of several different types [a similar portmanteau approach to justification has been taken by Mello in her analysis of vaccine injury compensation (Mello 2008)].

If these arguments go through they also provide strong support for a societal obligation to put into place legislation and social policies aimed at preventing some of the costs and burdens from occurring, e.g. by protecting the employment rights of people in isolation, quarantine or complying with recommend social distancing as well as the rights of their relatives, and by working to limit the stigmatising effects of being an asymptomatic carrier.

The pragmatic arguments only need to be stated briefly here, since they have already been analysed in detail by Rothstein and Talbott (2007b) and Ly et al. (2009). They are based on the assumption that if people are threatened with big losses such as loss of livelihood or significant risks to health if they comply with public health interventions, then compliance will be undermined and it will become more likely that they will try to evade the public health intervention. This is in essence an argument based on the incentive structure created by, for instance home quarantine or self-isolation without protection of employment status and compensation for lost wages. If compliance is a desirable societal goal it can then be achieved by changing the incentive structure in either of two ways. We can either increase the penalties for non-compliance or decrease the costs of compliance, or some combination of the two. Unless non-compliance is easily detectable and a penalty approach therefore likely to work, decreasing the costs of compliance, for instance through compensation schemes becomes the pragmatically preferable policy option. Elements of the Chinese response to the Covid-19 outbreak illustrates that effective surveillance and punishment regimes may be possible (Culbertson 2020), but probably only in societies that already have a high level of state surveillance.

There are also several ethical lines of argument supporting an obligation to compensate. Apart from consequentialist arguments which will broadly track the pragmatic considerations outlined above and support compensation because it leads to more effective public health interventions and better aggregate outcomes, ethical arguments can be based on^6^:

- A perfect duty of non-maleficence
  - A duty to compensate when causing harm
- An imperfect duty of beneficence
- Justice
- Reciprocity (in itself or as part of a solidarity based consideration)

Let us analyse these in turn. It is plausible that individuals have a perfect*prima facie* duty not to harm others and that the state has a similar perfect *prima facie* duty not to cause harm. The assumption that the public health interventions we discuss here are legitimate entail that this perfect *prima facie* duty has been considered as part of the decisions to implement the interventions and that it has been found that it is outweighed by one or more other *prima facie* duties that are more important in the context of the specific decision, e.g. the state’s duty to protect the life and welfare of its citizens. But, the fact that the harms and losses caused by the intervention are justified in the sense that they are not sufficiently severe to show that we should not implement the intervention does not in itself show that it would be justified to ignore these harms and losses and the effect they have on those people to whom they fall. The duty not to harm come with a correlative duty to make good any harm that we do cause, for instance by compensation.^7^ That duty is also a *prima facie* duty and can be outweighed by other duties. However, when analysing whether the duty to compensate for harms done is outweighed the question is no longer whether the public health intervention, with the known imposition of harm is justified, but which of two possible interventions is most justified, i.e. the intervention with or the intervention without a compensation scheme. In that analysis and weighing the state’s duty to protect the life and welfare of its citizens will in many cases have much less weight, simply because a compensation scheme does not impede the state’s ability to discharge that duty.

The general imperfect duty of beneficence also supports providing compensation in this context, at least in cases where the harms and costs lead to a significant reduction in welfare. Here the consideration would not be the causal connection between the public health intervention and the harms and cost, but simply the claim to help generated by a loss of that magnitude.

What about considerations of justice? Decisions about when and how to implement public health interventions raise basic issues of justice, including fundamental issues about distributive social justice. The issue of whether justice arguments support compensation is, however a more restricted question about whether those whose positions are changed in a negative way by the intervention should be compensated as a matter of justice. Or to put it differently, do the harms, costs and burdens caused by public health interventions count as unjust changes that have to be rectified? This focus on negative change significantly limits the range of approaches to justice we need to consider. One way of approaching this question is through the lens of luck egalitarianism (Arneson 2004; Lippert-Rasmussen 2015). Are these harms, costs and burdens the result of brute or option luck? Becoming the target of a public health intervention is in most cases the result of a complex causal network where the individual in question has little or no control over many of the causal nodes. For instance, most MRSA is either picked up in hospitals or other health care settings, or from animals in farming; and it is eminently possible to pick up MRSA and become a carrier even when taking all the recommended precautions. Similarly complex causal networks lies behind individual cases of flu during a flu epidemic etc. In all of these causal networks there will be some nodes that reflect personal choices, some that reflect the choices of others, some that reflect structural background conditions, and some that simply reflect bad luck, and many nodes will even in a single node reflect a complex combination of all four. E.g. Let us imagine that ending up sitting next to an infectious flu sufferer on the bus going home from work at Manchester University is the causal node which constitutes the Mackiean INUS^8^ factor that determines that a specific person becomes infected. This may be the result of a choice to use public transport, the choice of someone else to come to work even when ill, the bus always being very full due to public transport funding issues, and making an unlucky choice between the last two empty seats. If this analysis of the causal situation is correct in most cases it means that acquiring the infection, or acquiring the possibility of having an infection which makes you a target for public health intervention is a question of brute luck and not a question of option luck, even though the causal network contains some nodes of personal choice. And, as such the costs that follow from this bad brute luck ought to be compensated.

Another justice based argument which should appeal to consequentialists and welfarists in general considers the public health intervention through the lens of Pareto optimality. Pareto optimality is the standard approach in economics to evaluating the acceptability of a social change. I have made this argument in an earlier paper, and cannot state it more clearly than I did then:

If detention in the public health context succeeds in limiting the spread of the disease it constitutes a potential Pareto optimal social change compared to the situation where the disease outbreak is allowed to run its course unchecked. Analysis of the Toronto SARS outbreak does for instance seem to indicate that despite the very large costs of quarantine the economic benefits outweigh the costs (Gupta et al. 2005). A potential Pareto optimal social change is by definition a change where some lose and some win but where there is a net social welfare gain such that the winners could compensate the losers for their loss. A potential Pareto optimal social change can therefore, again by definition, be converted into an actual Pareto optimal change, i.e. a change where some gain welfare but no one loses welfare, if the winners compensate the losers for their loss.

Let us assume (1) that the number of detained persons is small compared to the general population and (2) that the principle of diminishing marginal utility of resources holds (at least approximately). Then it follows straightforwardly that the social state after compensation has been provided will have higher aggregate welfare than the state before compensation. The former losers will gain more welfare from the redistribution inherent in compensation than the former winners will lose, and the net loss to each former winner will be small.

There is thus a strong welfarist argument for providing compensation. This argument is even stronger if we accept a form of prioritarianism because this will direct us to give special weight to welfare improvements for those who are worst off in welfare terms (Rabinowicz 2001). (Holm 2009, p. 201).

Reciprocity is, finally the basic idea that there is an obligation to respond in a proportionate and fitting manner for the good or ill we receive from others (Becker 1986, 2005). We can think of reciprocity as a free standing obligation or as a part of distributive justice or a larger framework of social solidarity. Within a social solidarity framework enacting reciprocity will be one of the solidaristic practices that sustain the overall solidarity within the relevant group or society.^9^ In the present context not much hinges on precisely where we locate reciprocity. The good involved in the present context is willingly complying with public health interventions that are instituted to achieve a wider public good. The proportionate and fitting response is gratitude towards those fellow citizens who comply (Silva and Viens 2015), and if compliance generates significant personal costs for them a proportionate and fitting response would be to help to defray those costs. While we may not call our response to the costs of our fellow citizens ‘compensation’ when contemplating it from the perspective of reciprocity, the end result is essentially the same. They have freely provided an important good for society and letting them carry the full costs themselves would not be a fitting and proportionate response.

Slightly more formally we can combine the pragmatic and the ethical lines of argument in the following way (where PHI = Public Health Intervention, PEmp is an empirical premise, PEth an ethical premise, PPrag a pragmatic premise and C a conclusion).

PEmp1: PHI carried out in order to ensure public benefit
PEmp2: The positive effects of the PHI are widely shared (and may in the technical sense constitute a public good)
PEmp3: The harms/burdens fall to a small number of people
PEth4: There is no particular justification for harming/burdening these specific people (e.g. they have not explicitly or implicitly waived their rights not to be harmed, they are not personally responsible for being in the state which necessitates the PHI etc.)
PEth5: The people who are harmed/burdened do not have any specific duty to accept this harm
PEth6: Harms and burdens caused by actions such as PHI should normally be compensated *(follows from a number of independent ethical considerations enumerated above)*
IC1: There is an ethical case for compensation.
PPrag6: The existence of a compensation system is likely to increase adherence to the PHI

C. The people who are harmed and/or burdened by the PHI should be compensated for the harm and/or burden.

This argument is valid, but it is obviously only sound in those cases where all the premises are true.

## What could defeat a societal obligation to compensate?

Even if we accept the argument above and therefore accept that there is good pragmatic and ethical justification to compensate people for losses they incur due to public health interventions such an obligation would not necessarily be absolute and we therefore need to consider what, if anything could defeat the obligation in general and in a particular context.

There are at least two possible general defeasors of an obligation to compensate (1) lack of resources, and (2) no obligation to compensate for losses when the required action is the discharge of a moral duty. Let us analyse these in turn.

The argument from lack of resources is, that whereas there may be an ideal obligation to compensate, this obligation does not have to be discharged when there are no available resources with which to compensate, since ought implies can. This is a strong argument in resource poor countries with very weak health care systems that cannot provide for even the basic health care needs of the population. It is, however a much weaker argument in richer countries where health care is provided for even minor ailments. In such countries there is no absolute lack of resources leading to an ‘ought implies can’ question, but simply a fairly standard question of resource allocation between different activities. We also need to note that where outbreaks of infectious diseases have potential cross border effects the pool of those who fall under an obligation to compensate broadens and may no longer be limited to the particular resource poor country where an outbreak started.

The argument based on the claim that we should not compensate people for losses they incur as part of doing something which is their moral duty sounds initially plausible. I have myself argued some time ago with John Harris that there is a strong moral duty not to infect others, or put them at risk of infection (Harris and Holm 1995). Such a duty can be defended on both deontological and consequentialist grounds, but we must note in passing that in the cases we are discussing here it is not in most cases a ‘duty of easy rescue’ (Singer 1972).^10^ It is in many cases not strictly speaking a ‘duty of rescue’ at all, since the duty involved is a duty not to cause someone to be worse off than they were before, because they now have an infection. It is not a duty to make someone significantly better off by rescuing them from some dangerous situation they are currently in. And, unless the losses incurred are trivial it is also a ‘duty of (at least) moderately costly rescue’ and it is often a duty where the eventual beneficiaries are unknowable individuals who are spared infection, and not any identifiable person in need of rescue *ex ante*. Giubilini et al. (2018) and Giubilini and Savulescu (2019) have recently argued that we can convert a duty of costly rescue to a duty of easy rescue in relation to isolation and quarantine by changing the conditions under which people are isolated and quarantined, including compensating for loss of livelihood. This is, however not completely convincing in relation to quarantine, partly because it still raises some thorny issues about how we compensate *ex post* for death as a result of quarantine (Holm 2018). However, even if we accept that there is a strong duty to reduce the risk of the spread of infection by complying with public health interventions, as I think we should, this does not in itself extinguish a claim for compensation for losses or the state’s corresponding obligation to provide such compensation. Fire officers have a moral duty as well as a contractual and professional obligation to rescue people from burning buildings. This is not an absolute duty and they are entitled to not attempt rescues when the risks of harm are too great. However, when a fire officer attempts a rescue and suffers harm during that attempt we do not say ‘We thank you for doing your duty. You are a hero! However, it was your duty and therefore you have to pay for the costs yourself and we will not compensate you for the harm you suffered.’ It would be distinctly odd to say this, just as it would be distinctly odd to say to someone who suffered vaccine induced polio that any claim to compensation is extinguished because there is a moral duty to participate in the vaccination program, or to say it to a person who lost her job by adhering to a recommended voluntary social distancing scheme and staying at home with her delightful potential super-spreaders (i.e. children).

We need to distinguish this argument from the analogue to *de minimis non curat lex* in this context. Some losses may be so small that they fall below the threshold for compensation. The pain of having an injection does create a small welfare loss, as does being stopped at the airport for a temperature measurement, but these welfare losses are so small and inconsequential that we are probably justified in ignoring them completely in standard cases.^11^

In particular contexts we have to consider whether personal responsibility for being in the group that is targeted by the public health intervention can defeat a claim to compensation for losses. Let us first look at the simplest case where the personal responsibility arises as the result of a deliberate action aimed at becoming a member of the group targeted by the intervention.

If someone had, for instance deliberately acquired an infection in order to be isolated, should that person be compensated for the losses caused by the isolation? In a perfect system of compensation this should not really be an issue, since the amount of compensation would be equal to the loss caused by the isolation and the person would therefore not be better off than if she had not acquired the infection. The losses caused by the isolation would be perfectly balanced by the amount of compensation and she would still suffer whatever welfare and other losses that followed directly from the infection. However, any actual compensation scheme is likely either to over- or undercompensate. If the system systematically undercompensates there would be no incentive to try to ‘exploit the system’ since any attempt of exploitation would lead to a loss and the problem would again not arise. It is only if the system overcompensates, or is believed to overcompensate that there would be an incentive created by the compensation to become part of the target group. If such situations actually occur we might decide not to compensate when there is clear evidence of deliberate acquisition of the infectious disease, although we might also consider that a person would have to be in a fairly deprived prior position if the acquisition of an infectious disease so serious that it warrants isolation is seen as an attractive choice.

The question of the role of personal responsibility arises more commonly in situations where the person has not deliberately aimed at becoming part of the group targeted by the public health intervention, but where some action or ongoing activity has significantly increased the risk of acquiring an infectious disease (or contact status) and thereby made the person a target of intervention. Let us first note that intuitions about whether such responsibility defeats a claim to compensation differ according to the characteristics of the action or activity creating the activity. Children are super spreaders of flu, but we would not normally think that teachers and nursery nurses diminish their claim to compensation because their job makes it more likely that they acquire flu than the average person since it brings them into contact with many children. This intuition is plausibly based on the underlying view that being a teacher or a nursery nurse is a morally laudable activity. We could probably generate the opposite intuition by describing a thrill seeker visiting an Ebola emergency hospital just to have a look and get the experience, since thrill seeking is not in itself a morally laudable activity. But, more generally we can also point to the causal analysis presented in the section of justice and luck egalitarianism above. Except in cases of deliberately acquired infection it is simply not correct to state, as if it was an objective fact that the choices of the person has caused the infection or contact status. Stating this is itself a normative choice to focus on only some nodes of the causal network and ignore others (see von Wright 1971 for a general account of the inevitable element of choice in causal attribution).

## Conclusion

In this paper I have argued that society has a strong *prima facie* obligation to compensate individuals who suffer non-trivial financial or non-financial losses as a result of being the subjects of targeted public health interventions aimed at controlling the spread of infectious diseases, and that it also has an obligation to put in place regulation aimed at minimising such losses (e.g. by protecting the employment of people in isolation, quarantine or complying with social distancing advice). It follows from the argument that compensation schemes should be based on no-fault mechanisms, and from the more general consideration that if society has an obligation to compensate it should also ensure that people can get the compensation they are due that a compensation scheme has to (1) be easy to access,^12^ (2) compensate everyone with a valid claim, (3) resolve claims quickly, and (4) provide adequate compensation.

The argument has been developed within the context of public health interventions aimed at controlling infectious diseases and it requires further research to determine to what extent it can be applied outside this context.

## Footnotes

1 The final version of the paper has been written in February 2020 during the Covid-19 outbreak. Any references to that outbreak are therefore to the state of knowledge at that time.

2 It is assumed that the public health intervention is implemented by an agency acting on behalf of the state.

3 This last assumption about effective access to redress and compensation for error, fault or negligence is not true as a statement of empirical fact. In many or perhaps even most jurisdictions obtaining redress and compensation is very difficult. However, it is still important to keep the issue of compensation for costs or burdens caused by error, fault or negligence conceptually distinct from the issue of compensation for costs or burdens that are created directly by the intervention when it is applied as it should be applied.

4 There may now be ‘digital nomads’ who are able to work in quarantine etc. if they have good WiFi, but their number is probably still so small that we do not need to analyse their situation separately.

5 The implications for health care staff who are identified as asymptomatic carriers may be much larger, but will not be specifically analysed here. But note that the general framework developed here entails that there is an obligation to compensate staff for losses that follow from necessary and legitimate restrictions of their activities prompted by their carrier status.

6 This does not pretend to be an exhaustive list.

7 Making good the harm caused may also involve other elements like providing an apology or an explanation.

8 INUS factor = Insufficient but Necessary part of an Uneccessary but Sufficient causal complex (Mackie 1974).

9 Viens et al 2009 provides a more general analysis of the role of reciprocity in justifying restrictive public health interventions.

10 The need to be clear about this distinction was pointed out to me in discussion by Tom Douglas.

11 Any half decent philosopher can construct a hypothetical case where *de minimis* losses are very frequent and very unequally distributed between people, so that some experience very many and others none at all. If such a situation actually occurred we could have obligations to compensate when the additive loss to an individual crosses a *de minimis* threshold.

12 Potentially providing compensation automatically as a matter of course when losses are easily quantifiable in relation to e.g. isolation, quarantine and social distancing. Making it difficult to access compensation will almost inevitably lead to a situation where compensation is inequitably distributed to the benefit to the detriment of the least resourceful claimants.
